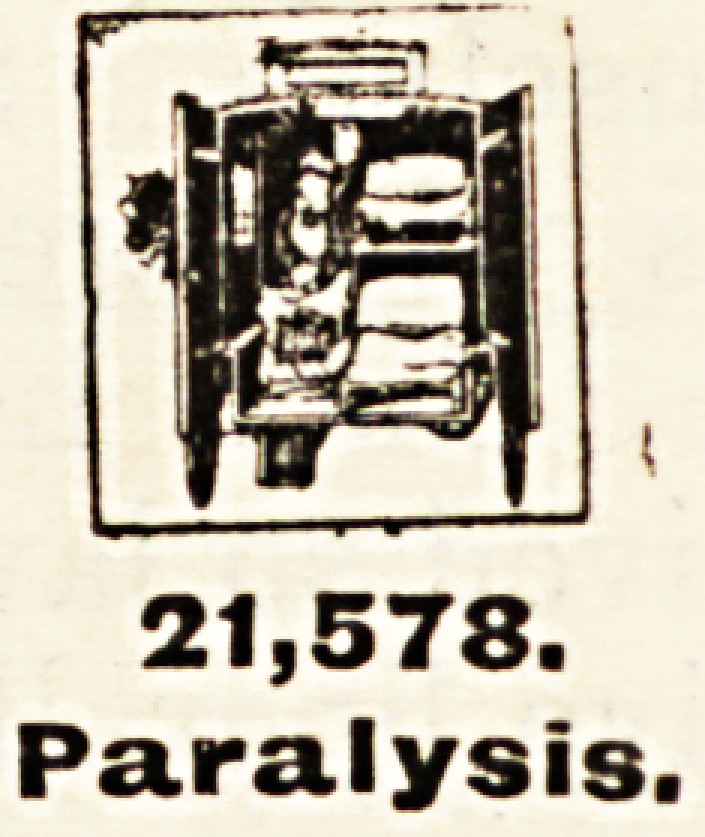# Special Hospital Sunday Supplement

**Published:** 1905-06-24

**Authors:** 


### Special Hospital Sunday Supplement.

### Wanted: One Hundred Thousand Pounds.

Ever since 1895 the active spirits on the Metropolitan Hospital Sunday Fund Council have believed it to be possible to raise £100,000 on Hospital Sunday. Previous to that date £60,000 was thought possible, but the larger amount was regarded by the majority at any rate as a dream rather than a possibility. In 1895, however, this journal organised a house-to-house distribution of its Special Hospital Sunday Fund Supplement of that year, and its editor wrote personally to the principal householders in the metropolis, with the result that the stationary and even falling tendency of the total receipts was stopped, and a record sum raised. The Council of the Sunday Fund in that year, in a special resolution, acknowledged "the " benefit that the Fund had derived from the " advocacy of its claims in The Hospital news- " paper, and by other means," "in obtaining sub- " scriptions raising the Fund to the unprecedented "sum of over £60,000." Encouraged by such a magnificent result several people were stirred up to take an interest in the Fund. Sir Savile Crossley and others continued or gave large individual donations, and in 1899 Mr. George Herring, whose portrait we published last year, contributed the magnificent sum of £10,000. Mr. George Herring gave a similar sum each year up to and including 1901. In 1902 when Sir Edmund Currie was appointed secretary to the Fund, Mr. George Herring made the generous offer to add five shillings to every pound raised in a place of worship on Hospital Sunday in that year, thus raising his contribution to £11,575. In 1903 he gave on identical conditions £12,302, and in 1904 £11,926, making together £65,803. So munificent a sum proves Mr. Herring's noble and generous spirit towards the hospitals, but his gifts and example have conferred far greater benefits on the Fund than the intrinsic value even these splendid contributions represent.

The permanent results of the influences we have just referred to are shown in the figures published by the Council of the Fund. In 1895 The Hospital's special effort raised the donations in round figures to £22,000, and the amount received from the congregations was raised to £38,370, the highest amount in any year with the exception of 1890, when it reached £38,823. In 1896, the congregations contributed £40,501; then they fell away during the next five years, but in 1902 they rose to £46,362, and in 1903 to £49,209, and in 1905 they should reach, if they do not even exceed, £50,000. These figures testify to the influence for good which has resulted to the Hospital Sunday Fund through the offer of Mr. George Herring to add 5s. to every pound raised by the congregations. It will be seen from the interview with Sir Edmund Currie, which we publish elsewhere, that this year Mr. George Herring has made another and further advance by consenting to add five shillings in the pound to every sovereign contributed to Hospital Sunday from whatever source it may come. Mr. George Herring is well known and widely respected in the City of London. His generosity to the hospitals through the Sunday Fund is gratefully recognised, and we believe that his friendly challenge to the City will stimulate many to contribute this year, for many givers must feel encouragement from the knowledge that 25 per cent. will be added to whatever amount they may each give to the hospitals. If the City subscribes £37,500, there is every prospect that the dream of former years may become a practical reality, for then the total receipts will reach £100,000.

It is a real advantage to the Metropolitan Hospital Sunday Fund that Sir Edmund Hay Currie should have devoted his whole time and energies to its work. His influence and its results may in some measure be gathered from the figures we have given above, but in these days the active, continuous, and whole-hearted efforts of one man in the cause of a great Fund like this must secure its recognition and stimulate many to exert themselves who might otherwise be half-hearted or even indifferent. Sir Edmund Currie this year, with his son's assistance, has paid a personal visit to some hundreds of the clergy and, the great majority of these gentlemen, for the first time, will issue a personal letter to those residents and members of their congregation who may be absent on Hospital Sunday, urging them to send some gift to the hospitals on that date. Canon Fleming, the Rev. Prebendary Ridgeway, and others have proved the value of efforts of this kind, which were originated by the late Canon Miller, the real founder of Hospital Sunday in these islands, who used to describe these letters as his "golden pheasants." Then, too, Sir Edmund Currie has rendered invaluable service by going carefully into the details of the expenditure of the hospitals, and so bringing out the facts for the consideration of the Distribution Committee, whereby the attention of the hospital authorities has been specially called, in several instances, to the figures in the accounts with the best results. A knowledge of these facts cannot fail to enlist the sympathy and support of men of business throughout the metropolis, who may be thus encouraged to increase the amount of their individual gifts, and to support the hospitals in larger measure through the Sunday Fund. Their adequate upkeep, which can alone guarantee the efficiency of the hospitals, is the business of everybody. We have this year purposely confined our appeal for the hospitals mainly to facts and figures, because they afford precise evidence to all thoughtful people that to-day the Hospital Sunday Fund is so organised and administered as to entitle it to the warm and generous support of every resident in the metropolis.

### The Origin of the Hospital Sunday Supplements.

It may be interesting to restate the circumstances which led the proprietors of The Hospital to publish its Hospital Sunday Fund supplements. A brief history of their origin will be found in The Hospital of June 22nd, 1895, page 205.

These receipts fell to £24,905 in 1878. They rose to £39,330 in 1884, when the contributing congregations numbered 1,522. By 1885 the total receipts had fallen again to £34,320, and in that year Dr. James Wakley, who was suffering from a mortal disease, sent for the writer, and urged him to devise some scheme which would help the hospitals by increasing the interest in and collections on Hospital Sunday. In the result a number of public meetings were organised throughout London, at which some of the most eminent leaders of public opinion kindly consented to speak. Dr. Wakley, as editor of the *Lancet*, published a special supplement which was edited by Mr. Burdett, and widely circulated. In the result the total receipts in 1886, although the congregations were less than 1,600, reached £40,393.

With a view to still further stimulate the interest of the public in the voluntary system of hospital support, Dr. Wakley and the present writer discussed together the outlines of a newspaper which should deal with all the questions involved by the maintenance of an adequate hospital system. In the result the first number of The Hospital was published on October 2, 1886. In the year 1887, following Dr. Wakley's death, the proprietors of the *Lancet* discontinued the issue of special supplements, and The Hospital filled the void thus created by publishing its first Hospital Sunday Supplement in that year, and has continued the practice ever since. In 1888 the *Lancet* again issued a special supplement, and the present proprietors, like those of The Hospital have continued to do so, and have circulated many thousands of abstracts from it amongst the congregations each year since that date. Such is a brief history of the origin of this journal and its special appeal supplements in aid of the voluntary hospitals and charities.

### The Past and Present of the Metropolitan Hospital Sunday Fund.

### INTERVIEW WITH SIR EDMUND HAY CURRIE.

With the hope of both creating and stimulating interest in the Metropolitan Hospital Sunday Fund, by placing before the readers of the special Hospital *Sunday Supplement* a brief account of the origin and present position of the movement, Sir Edmund Hay Currie was invited to give me the information necessary for the purpose. Apart from the fact that he is now Secretary of the Fund, there is no man living who is better acquainted with its history than Sir Edmund, for he has been associated with it from the outset, and is largely identified with its successful development. Possessing the advantage of a complete knowledge of hospital work gained in a period of half a century, and commencing when he went fresh from Harrow to Scutari, his experience as Chairman for many years of the London Hospital, the Poplar and Stepney Sick Asylum, and Vice-chairman of the Metropolitan Asylums Board, renders a task which would be extremely difficult to the uninitiated comparatively easy, while his love for the work, of which he has so repeatedly afforded practical proof, supplies a motive power the value of which it is impossible to exaggerate.

"You want to know how we started?" said Sir Edmund, when I saw him at the office of the Fund. "Well, it is more than 30 years since the movement was mooted. The first meeting took place at the London Tavern on the 21st February, 1872, when a number of gentlemen associated with London hospitals met together in order to form a committee. It should, however, be said here that the credit for the origin of the movement belonged to the Rev. Dr. J. C. Miller, then Vicar of Greenwich, but previously Rector of St. Martin's, Birmingham, where a similar Fund, of which he was one of the creators, was already in existence."

"Can you give me the names of those who were present at that historic meeting?"

"Mr. R. B. Martin, M.P., the Chairman of St. Mark's Hospital; Mr. T. Christy, Trustee of St. Peter's Hospital; Mr. James Hopgood, Chairman of the Royal Free Hospital; Mr. R. A. Outhwaite, Secretary of the City of London Lying-in Hospital; Mr. H. J. Kelly, Secretary of University College Hospital; the Rev. J. F. Kitto, Secretary of Poplar Hospital; Mr. W. R. Scott, Honorary Secretary of St. Peter's Hospital; Mr. J. S. Fry, Chairman of the Metropolitan Free Hospital; Mr. H. Sykes Thornton, Treasurer of Brompton Hospital and of the Hospital for Sick Children in Great Ormond Street; Mr. W. J. Nixon, Secretary of the London Hospital; Mr. Jabez Hogg, of the Westminster Ophthalmic and Charing Cross Hospitals; Mr. J. S. Gilliat, of St. Peter's Hospital; Captain Palliser, of the Cancer Hospital; Mr. W. F. Ainsworth, Treasurer of the West London Hospital; Mr. C. Lowther Kemp, Secretary of the Chest Hospital, City Road; Mr. Guy Scudamore, Secretary of the Samaritan Free Hospital; Mr. Peter Gowlland, Senior Surgeon at St. Mark's Hospital; Mr. J. A. Johnson, of the North London Hospital for Children; Mr. Vincent Yardley, Secretary of the British Lying-in Hospital; Mr. E. S. Norris, Treasurer of the East London Hospital for Children; Mr. J. J. Thompson, Honorary Secretary of the Victoria Hospital for Children; Mr. S. S. Smith, Secretary of the Hospital for Diseases of the Skin; and myself in my capacity of Chairman of the London Hospital."

"What action was decided upon before you separated?"

"It was determined to form a committee, after a resolution, moved by myself and seconded by Mr. Christy, had been carried unanimously. The resolution was to the effect that in the opinion of the meeting it was desirable that the principle of Hospital Sunday be adopted."

"And when did the Committee meet for the first time?"

"On November 27th, 1872. They then deputed

Mr. Martin and myself to wait upon the Lord Mayor of the year, Sir Sydney Waterlow, asking him to preside at a meeting in the Mansion House preliminary to a representative conference. This meeting took place on December 16th, 1872, was presided over by Sir S. Waterlow, as chief magistrate, and was of a very influential character. Among those present were the Archbishop of Canterbury and Canon Miller. Dr. Brock, a well-known Baptist minister, moved, and Archbishop Manning, as he then was, seconded, a proposal to the effect that the Lord Mayor be requested to issue letters of invitation to representative clergy of the various religious denominations, asking each of them to request a layman of their church or chapel to meet the Committee at a conference in the Egyptian Hall on the Hospital Sunday question."

"Was there another meeting before the Conference?"

"Yes; on Friday, January 10th, 1873, the Committee met, and having come to the conclusion that the success attending Hospital Sunday collections in provincial towns justified the belief that the movement would be equally successful in London, they passed a resolution in favour of a simultaneous annual collection being made at all places of worship in the metropolis on behalf of the hospitals and medical charities. This resolution was proposed by myself and seconded by Dr. Miller. It was also decided that a representative council, with power to make arrangements for the collection, should be formed."

"How was the council composed?"

"Of twenty-five clerical and twenty-five lay members, the Lord Mayor for the year being appointed permanent President of the Fund. A resolution was next passed giving the Council authority to appoint a central committee of distribution, unconnected with hospitals, whose decision should be final."

"Did you also settle at that meeting the principle of the system of distribution?"

"We did, and it was, as it stands to-day, based upon the average ordinary expenditure of each institution for the last three years, after deducting therefrom the income derived from endowments, realised property, and legacies exceeding £100. It was also determined that no institution should be permitted to participate in the distribution of the Fund whose cost of management had been ascertained by the Committee to exceed a reasonable percentage of the whole amount expended."

"Were the Distribution Committee elected then?"

"No; the next step was the Conference at the Egyptian Hall on January 16th, 1873. Earlier in the day the Committee met, and Dr. Brock, Dr. Kennedy, and Archbishop Manning having handed in lists of representative clergymen and ministers of their churches, the Lord Mayor presented a similar list drawn up by the Archbishop of Canterbury. At the Conference itself, Canon Miller proposed, Dr. Kennedy seconded, and Sir Anthony de Rothschild and the Rev. Dr. Rigg supported a resolution identical with that adopted by the Committee on January 10th. It was unanimously adopted, and the simultaneous annual collection having been agreed to, Bishop Claughton proposed, Archbishop Manning seconded, and the Rev. P. W. Clayden supported a motion for the appointment of a representative council, the 50 clergy and laity suggested by the Committee being unanimously elected."

"When did the Council first meet?"

"On January 31st, 1873. A number of well-known men were present, and at that meeting Archbishop Manning proposed, and the Rev. Dr. Allan seconded, that Mr. R. B. Martin and myself should be appointed joint honorary secretaries to the Fund. My connection with it has been unbroken from that date."

"How was the first Distribution Committee constituted?"

"The Council met on February 17, 1873, and elected Sir Sydney Waterlow, Sir Anthony de Rothschild, Mr. Samuel Morley, M.P., Mr. W. H. Smith, M.P., Lord Mahon, M.P., Mr. Jervoise Smith, Mr. Thomson Hankey, Mr. W. McArthur, M.P., and Mr. Martin and myself (*ex-officio*), the 10 members of the Committee."

"The future of the movement depended very materially upon the Distribution Committee?"

"They were the pivot upon which almost everything turned. At the outside their lines of working were clearly fixed in accordance with the resolution passed by the General Purposes Committee, but the needs and merits of every particular institution have always been considered. For example, an award is never either reduced or withheld until after opportunity has been afforded for an explanation to the managers of the hospital in question."

"How many congregations contributed to the Fund at the first collection in 1873?"

"A thousand and seventy-two, the amount collected being £25,855 13s., or with legacies, special donations and dividends £27,700 8s. 1d."

"And since then the progress has been steady and continuous?"

"Practically; though there have been fluctuations. But the great point is that last year, the thirty-second after the foundation of the Fund, the number of congregations contributing was 2,004, and the amount collected £47,911 14s. 2d., or with legacies, donations, and dividends, £63,054 17s. 8d."

"How many grants did you make in 1873?"

"A hundred and five. Last year the number was 218."

"Now, will you tell me, please, what is being done in order to augment the collection of 1905?"

"First, I think that reference should be made to an enlargement of the munificent offer of Mr. George Herring. As most people are aware, having given us £10,000 for several years, in 1902 Mr. Herring offered the alternative in future of £10,000 or 5s. in the pound for every pound contributed by the places of worship up to £25,000. Hitherto we have only been able to claim less than half this amount. But I called on Mr. Herring the other day, and, having pointed out that, in my judgment we are nearly, if not quite, at high-water mark and can scarcely hope to exceed £50,000 from the churches and chapels in London, I asked him whether, if I initiated a supplementary collection in the City of London proper, he would be good enough to extend his limit and regard the sum accruing from the supplementary collection in the

City as a part of that contributed by the places of worship."

"What did he say?"

"I have just had his reply. Mr. Herring kindly agrees that so far as the City of London is concerned, money so returned in answer to a special appeal shall be considered as having been donated in a place of public worship. We are, therefore, at very short notice, making this year a special appeal to the City."

"Assuming the collection in the places of worship to be £50,000," continued Sir Edmund Currie, "and supposing, as we suppose and hope, that another £50,000 can be raised in the City of London by donations, the Distribution Committee of the Sunday Fund will be able, thanks to Mr. Herring's generosity, to distribute up to £125,000, which would suffice to relieve the pressure for the maintenance and support of the patients in London hospitals to an immense extent."

Observing that I had noticed on the wall of the room a map of the ecclesiastical parishes of London marked with red and black flags, Sir Edmund said: "This year I have visited by appointment 160 incumbents of London churches, extending from Hampstead to Streatham and from Ealing to Greenwich, with the outlying contributing congregations in Bromley, Beckenham, Chislehurst and Reigate. These parishes are marked on the map with red flags. The committee have allowed me to have the assistance of my son in the secretarial work which I undertook at the suggestion of Sir Joseph Dimsdale four years ago, and he has visited the incumbents of the parishes marked with black flags. We have asked all these vicars to send a letter, with a penny stamp upon it, not only to the members of their congregations, but also to all the parishioners capable of contributing, asking them to send something to their own place of worship."

"Was that your own idea?"

"It was suggested to me by the statement of the vicar of a rich West End parish that many of his wealthiest parishioners are only in London for a few weeks, regard the clergyman in their country parish as their vicar, and do not attend his church on Hospital Sunday. He added that it was a matter of comment that in some other parishes not more wealthy than his own the collections were much larger, and the difference was really due to the number of residents in the latter of rich City merchants."

"How has the suggestion been received?"

"It has met with a hearty response. For instance, Dr. Carter, the vicar of St. Dionis, Parson's Green, Fulham, has sent a signed letter to a thousand of his parishioners, in which he strongly urges those who cannot attend their ordinary church or chapel to forward an offering on or before Saturday, June 24, and says, 'This is quite an undenominational fund, so I have no hesitation in asking all of you to help, even if we do not, unhappily, all see alike in matters of doctrine or worship.'"

"Some of the congregations are exceptionally liberal?"

"Yes; for more than 20 years Canon Fleming, of St. Michael's, Chester Square, has sent in over £1,000. He started in 1873 with £138, and last year the amount was £1,382. I believe he writes personal letters for a long time before the collection. Then there is Prebendary Ridgeway, of Christ Church, Lancaster Gate. In 1885 the sum sent in from this church was £519; last year it was £1,520 11s. 6d. The collection at St. Peter's, Vere Street, of which Canon Page Roberts is incumbent, is another remarkable instance. He began in 1878 with £45, and last year he sent in £644."

"Have you any illustrations of liberality on the part of Nonconformists and Roman Catholics?"

"For many years there was no collection at the City Temple, but last year Mr. Campbell sent in £300, which he said was only a beginning. Mr. Voysey, of the Theistic Church, has increased from £37 in 1882 to £318 in 1904; Rev. A Fleming, St. Columba, Pont Street, £206; Rev. Hardy Harwood, Union Chapel, Islington, £201; Rev. R. Roberts, Westbourne Grove, £125; Rev. R. Fotheringham, Blackheath, £115; Dr. Horton, Hampstead, £107; Rev. A. Connel, Regent Square Chapell, £100; Mr. Freeston, of the Unitarian Church, Kensington, £206, and Brompton Oratory headed the list of Roman Catholic Churches with £56. The two Jewish congregations ran each other very closely last year, the amount from the Great Synagogue being £255, and from the West London Synagogue £253."

"Do the ministers of religion write you often for surgical appliances and convalescent hospital letters?"

"I have a hundred letters more or less every morning asking for appliances. The committee are allowed to expend up to 5 per cent. of the amount collected on surgical appliances at the request of the clergy. We seldom contribute the entire sum required by an individual, but if we give a substantial

### HOSPITALS AND THEIR SPECIAL NEEDS.

**British Home and Hospital for Incurables, Streatham, S.W.**—£10,000 at once to repay banker's loan and to enable the work to be continued.

**Cancer Hospital (Free), Fulham Road, S.W.**—£4,000 to make good deficiency in income; £1,500 to carry out necessary sanitary alterations; £1,000 to maintain Pathological Department.

**Central London Ophthalmic Hospital, Gray's Inn Road, W.C.**—Funds to rebuild, as urged by King Edward's Hospital Fund, present accommodation being unsuitable.

**Chelsea Hospital for Women.**—Further contributions to the Emergency Fund, which is designed to guarantee a continuance of financial stability.

**City of London Lying-in Hospital, City Road, E.C.**—£35,000 for rebuilding; £1,000 to meet extra expense of temporary in-patients' department.

**East London Hospital for Children.** — £1,000 must be collected by June 30th, in order to secure further grants of £3,500.

**Guy's Hospital.**—£100,000 to pay debt, and £15,000 additional income.

**Great Northern Central Hospital, Holloway Road, N.**—£10,000 needed annually.

**Hospital and Home for Incurable Children, North Court, Hampstead, N.W.**—Greatly needs help to meet outlay involved in acquiring new premises.

portion there is no difficulty in getting the instrument-maker to take the rest in small instalments. With regard to the letters for the 30 convalescent homes to which we make grants, half the quantity given to ordinary subscribers are sent to us, and these are distributed to the clergy on application, not to persons needing rest, but places of recovery from illness, or after operation. We make a point of answering all applications by return of post, so that there is no vexatious delay."

"None of the money contributed is spent on bricks and mortar?"

"Every farthing goes to the maintenance of patients. The Distribution Committee rejoice to know that the cost of patients, possibly owing to their own care in going into all details most scrupulously, has been diminished year by year. Our grants, it may be added, are made not merely to London hospitals, but also to hospitals out of London treating London patients, and including 22 cottage hospitals, where much valuable work is done, as well as to no fewer than 58 dispensaries."

"Is it your opinion that the King's Fund has in any way operated to the detriment of the Hospital Sunday Fund?"

"On the contrary, it has had quite the reverse effect. It is most encouraging that while enormous sums have been raised by the King's Fund since it was started in 1897, the Hospital Sunday Fund, thanks to the clergy, who are the foundation, has gone on increasing."

"But you do not, nevertheless, consider the amount contributed to the Hospital Sunday Fund as large as it ought to be?"

"I am sure that it does not at all represent the amount which should, and I think would, be given if only we could reach all those who derive their income from property in the metropolis, but are not in London on Hospital Sunday. The total reached is apparently large, not really. Perhaps we should get a little more in legacies if we did not spend the money we receive at once. But it is a principle of the Fund to divide all that is sent in as quickly as possible, and within a few weeks of the collection on June 25th the whole of the money forwarded to us will be distributed amongst the hospitals and medical charities whose claims are recognised."

### The Service of Hospitals to the Community.

The direct and immediate value of hospitals in the scheme of national organisation is so manifest and obvious that it excites neither dissent nor discussion. Even the most severe critic of the various forms in which charity loves to display itself will allow that provision for the consequences of serious accident or illness, when these fall on the poorer members of the community, may be made without infraction of the laws of political economy. The experience is an every-day event which shows that in numberless individual cases it is through the hospital that the emergency of a dangerous accident is successfully met, that the acute disease is checked, that the bread-winner is restored hale and well to wife and family, and that those who are ready to perish find succour and new life. No one who knows the poorer quarters of our large towns can fail to be impressed by the almost unqualified confidence which the people place in the neighbouring hospital. For them it is a home of refuge in the darkest and most trying days of their experience. On these grounds alone the hospitals may well appeal to the more favoured members of the community for assistance and interest and service. They afford an opportunity for the practice of pure and genuine charity, disciplined by method and purpose, and unattended by demoralisation of the recipients.

There are, however, other and more direct claims which hospitals may present to members of the public whose financial position places them beyond the chance of charitable medical relief, and it is with these that we are here principally concerned. In the first place, it may be remarked that it is due to the existence of hospitals that the family medical practitioner has attained efficiency and skill in the practice of his profession. Only by the collection together of a considerable number of patients in an institution devoted to the care of the sick and the cure of disease could medical education in the modern sense of the term be successfully conducted. In this way the hospitals carry priceless benefits to numbers who never enter their walls, for they place a high level of medical and surgical skill and knowledge at the disposal of every household. It is the steady training of the student in hospital wards which makes possible the skilful and trusted

### HOSPITALS AND THEIR SPECIAL NEEDS.

**Hospital for Sick Children.**—Funds for current requirements. £1,000 will endow a bed in perpetuity.

**Hospital for Women, Soho.**—In want of immediate support for general purposes.

**Hospital for Gentlewomen, 90 Harley Street, W.**—Affords gratuitous medical treatment. Requires increase of £800 annually in voluntary support.

**King's College Hospital.**—A large sum required for the removal of the hospital to South London. Funds for current expenses.

**London Fever Hospital.**—Much in want of donations and subscriptions.

**London Temperance Hospital, Hampstead Road, N.W.**—£15,000 to build out-patients' department.

**London Homœopathetic Hospital, Great Ormond Street, W.C.**—A debt of £14,000 burdens this hospital.

**London Lock Hospital, Harrow Road, W.**—Money to meet current expenses. Much in need of annual subscriptions.

**Metropolitan Hospital.**—£5,500 owing to tradesmen. This hospital experiences great difficulty in obtaining support, owing to its situation in the midst of poverty.

**Middlesex Hospital, W.**—Money to replace large capital outlay on structural improvements and extensions.

**Miller Hospital.**—£2,500 to admit of the urgently demanded addition of 20 beds.

family medical adviser. Thus it may be truly said that every person who enjoys the benefits of good advice and efficient treatment when suffering from accident or illness is a debtor to institutions to which, perchance, he offers no meed of recognition.

But the hospital exercises yet other influences on medical and surgical attainments and possibilities. Members of the staff, in consequence of the special experiences and opportunities they enjoy, attain an exceptionally complete and confident knowledge of the processes of disease and of the methods by which these can be successfully treated. They become acquainted with disease in its more unusual and complex manifestations, and in this way are fitted to distinguish and deal with similar events when these occur among private patients. Hence in many instances these gentlemen are recognised by their fellow practitioners as competent consultants in cases of exceptional difficulty and anxiety, and thus the special and peculiar knowledge gained as a result of hospital practice comes to the bedside of the private patient and is placed at his service in his hour of difficulty. It is the fact of the hospital which makes such help so readily and generally accessible. In this respect it is worthy of note that such a consideration should appeal more particularly to those whose incomes may be fairly described as moderate rather than considerable. The rich man in his hour of trial will always be able to command the best medical or surgical assistance which exists, independent of distance and independent of expense. But for many, financial limitations forbid such a position. To these—and they form a large number—it is of high moment that the best aid and guidance can be obtained at a moderate outlay. And it is not too much to say that at the present day in this country there is no place so remote from physicians and surgeons of the very first order but that their presence can be secured for a very moderate fee. The explanation is found in the existence of the hospitals. It is principally within the walls of these institutions that is gained that special equipment which may so easily be secured for the benefit of the individual sufferer who is the victim of some special complication or difficulty. Here, again, the private patient, who, perhaps, knows little of hospitals or of their work and difficulties, accepts, it may be without thinking of the matter, service and benefit from their hands. The relationship between the hospitals and the community has still other aspects. It is not only, as already suggested, that hospitals secure the efficient training of the family practitioner, and give an experience which is of enormous value to the public in difficult cases. In addition, they make possible the advance of medical and surgical science and practice. New methods of diagnosis and new forms of treatment are suggested by the special circumstances of hospital work, and opportunities exist by which such suggestions may be practically tested. It is true that in a few instances some happy inspiration or the chance of a special experience has in private practice opened the way to some new development. But for the most part such developments find their starting point and their completion where opportunities for repeated and abundant observation exist. To take a single example, it is practically certain that but for the special sphere of hospital work the antiseptic system of surgery would never have been developed. And in the benefits of that system almost every person who has undergone a surgical operation during the last generation has enjoyed a substantial, and, possibly, an unrecognised, share. This is, doubtless, the most conspicuous illustration of the truth that the achievements of medicine and surgery rendered possible by hospitals pass to the service of the general community, but on a smaller and more limited scale parallel events are constantly occurring. The stimulus and demand of hospital work bear useful fruit in the shape of improved plans and methods and in enlarged knowledge. These arise and are tested in the practice of some individual physician or surgeon; they are repeated and perhaps improved by his colleagues; and introduced to the students in their day and generation, they are by these, as medical practitioners, carried into the homes of the people of all ranks and degrees. Thus, what was at first a special and peculiar advance in a particular sphere of hospital practice becomes shortly a commonplace of the profession and at the service of both rich and poor through the length and breadth of the land.

It is these and other such considerations which entitle us to claim that not merely on account of their primary and direct purpose are our hospitals worthy of support, but also because through them the sick and suffering of all classes gain the medical skill and assistance which bring relief in humanity's greatest need.

### HOSPITALS AND THEIR SPECIAL NEEDS.

**Mount Vernon Hospital for Consumption (Open-air Treatment), Hampstead, N.W.**—£100,000 to open 110 empty beds and pay off a loan of £15,000.

**National Hospital for Paralysed and Epileptic, Queen Square, W.C.**—£5,000 to make good deficit between maximum reliable income and lowest possible expenditure.

**North-Eastern Hospital for Children.**—Immediate help for current purposes and £8,700 to pay debt on new building.

**Paddington Green Children's Hospital, W.**—Owes its bankers £2,000; deficiency in reliable income of over £3,000.

**Queen Charlotte's Hospital, Marylebone Road, N.W.**—£10,000 to extend accommodation for constantly increasing number of patients and to enlarge nurses' home.

**Royal Orthopædic Hospital.**—About to amalgamate with National Orthopædic Hospital, and incorporate by charter. Funds for building on new premises required. Temporary address, 55 Bolsover Street, W.

**Royal London Ophthalmic Hospital.**—£50,000 towards Centenary Fund. Further support to meet annual expenses.

**Royal Waterloo Hospital for Children and Women, Waterloo Bridge Road, S.E.**—£50,000 for rebuilding and increase of income to £15,000 a year.

### A WORD TO LIVING LONDONERS.

### WAYS AND MEANS.

YEAR by year in our Special Hospital Sunday Supplements we have given statistics to show the proportion of the money given for the care of the sick in London by (1) the living, namely, the present inhabitants of the Metropolis, (2) by deceased benefactors and (3) by the patients themselves. We have pointed out how inadequate is the sum contributed by the living to insure the good work which is done by our hospitals being maintained. The latest complete returns, although slightly better than those given last year, are not calculated to make us particularly satisfied with ourselves. We find that in 1903, including St. Bartholomew's Hospital, the voluntary contributions amounted to 8s. 9d. in the pound, which, added to the 1s. 5d. received from the patients themselves, brings the amount given by the living up to 10s. 2d. in the pound, a slight increase on the amount received in 1902. When, therefore, Londoners are inclined to feel proud of their exceeding liberality to the hospitals they deceive themselves. That is a mean and contemptible spirit which takes credit for virtue it does not possess. If no such reproach is to rest on London this year it is imperative that all classes should give more liberally to the Hospital Sunday Fund and persuade others to follow their example.

### THE INCOME AVAILABLE FOR THE WORK DONE.

In the year 1903 hospital treatment was provided for about two millions one hundred and fifteen thousand persons, exclusive of the patients treated at the hospitals of the Metropolitan Asylums Board, and the total *ordinary* income and legacies received by the London voluntary hospitals and dispensaries for this purpose was £1,150,536, which was derived from the following sources:—

| | | |
|---|---|---|
| Charitable or voluntary contributions ... ... ... | £501,825 | or 44 per cent. |
| Income from invested property . | 258,788 | ,, 22 ,, |
| Legacies ... ... ... ... | 309,093 | ,, 27 ,, |
| Patients' payments ... ... | 80,830 | ,, 7 ,, |

So far as the above figures refer to St. Bartholomew's, Guy's, and St. Thomas's Hospitals, they have been confined to that portion of the revenue which is applicable to hospital purposes.

### HOW THE MONEY IS PROVIDED.

The mere statement that such and such an amount of money has been raised from one source or another does not, as a rule, greatly impress people. It is rather the difficulties which attend the raising of it, and the consideration of the sources from which it comes, that enable us to judge whether those who are in a position to give are doing their duty. It is therefore of the first importance that everyone should thoroughly understand where the money comes from to pay the cost of the relief given by the hospitals to the inhabitants of London, and to make this as clear as possible we have prepared diagrams, each representing a hand and a coin, which have been drawn to scale to show the proportion contributed out of every sovereign (1) by the living, *i.e.* those who receive the benefits, and (2) by deceased benefactors, many of whom have not only left their money to enable the good work to be carried on, but were also during their lives active workers for the hospitals. With a view to clear-

THE DEAD HAND GIVES 9S. 10D. OUT OF EVERY £1 RECEIVED BY THE HOSPITALS.

ness the diagrams have been drawn to represent the proportion of every sovereign given in 1903 by (*a*) the dead, (*b*) the living, and (*c*) the patients themselves. *The black hand* and the coin held by it represent the contributions from those now dead; *the white hand* represents the charitable contributions from the living, *i.e.* ourselves, the living Londoners; and the smallest coin indicates the amount received from patients' payments.

### HOSPITALS AND THEIR SPECIAL NEEDS.

**St. George's Hospital.**—£15,000 to make good deficiency in ordinary income.

**St. John's Hospital for Diseases of the Skin, Leicester Square, W.C.**—£8,000 still required for new out-patients' department.

**St. Mary's Hospital, Paddington.**—The demand for £60,000 is urgent to avoid closing wards.

**St. Saviour's Hospital, Osnaburgh Street, N.W.**—Further financial help, to continue the increasing work.

**Samaritan Free Hospital for Women and Children, Marylebone Road, N.W.**—£10,000 still required for new buildings and alterations. Funds for current expenses.

**Seamen's Hospital Society, Greenwich, S.E.**—Stands in great need of support to maintain the hospital and its branches at the Docks. This Society may hold real estate.

**University College Hospital, Gower Street, W.C.**—Funds to furnish new buildings, and an additional £5,000 annually to carry on increased work.

**West End Hospital for Diseases of Nervous System.**—Help to procure a convalescent home by the sea. Entirely without endowment.

Of every sovereign received 9s. 10d., or nearly *one half, is derived from legacies and the interest on gifts* from deceased benefactors, which have been invested in approved securities; 8s. 9d. out of every sovereign has been *given in charity* by the

**The Living gave**

**8s. 9d. in the £1.**

THE LIVING, *i.e.* THE PRESENT INHABITANTS, GIVE 8s. 9d. OF EACH £1 RECEIVED BY THE HOSPITALS.

present inhabitants of London, that is, *the living, for the benefit of whose generation the hospitals exist*; and 1s. 5d. of every sovereign has been contributed by those *who have been actually under treatment in the hospitals.* Let us study these figures carefully, and understand why they are so unsatisfactory, why they do not inspire us with confidence. It will be seen that a considerable portion of the 9s. 10d. given by the dead hand is derived from the legacies received during the year—to be exact, 5s. 4d. of it—and this source of income must always be fluctuating. As compared with 1902 there is a decrease of £30,000 in the amount received from legacies, against an increase of a similar amount when 1902 is compared with 1901. In 1896 also there was a sudden drop of £40,000 and in 1900 one of over £70,000. So the sum given by the dead hand may fail us sometimes, and this should be recognised by the living. A fact like this brings us face to face with the certainty that, unless such deficiency is made up by the increased gifts of the living, the great work of our London hospitals must be seriously crippled.

Now let us see how the gifts of the living compare this year with last. The actual proportion received is larger than in either 1900, 1901 or 1902, and represents 8s. 9d. in the sovereign as against 8s. 2d., 8s. 7d., and 8s. 5d. in these three years, but it must not be forgotten that even though it has increased slightly, were it not for the legacies received each year the income of the voluntary hospitals would be only equivalent to about 15s. or 16s. for every sovereign which the managers of those institutions have to expend on the service

**Patients' Payments yield**

**1s. 5d. in the £1.**

PATIENTS' PAYMENTS SUPPLY 1s. 5d. OF EACH £1 RECEIVED BY THE VOLUNTARY HOSPITALS.

of the sick and suffering in the metropolis. The amount received from the patients themselves is 1s. 5d. in the £1, as against 1s. 5d. in 1902 and 1901 and 1s. 7d. in 1900.

### The Meaning of the Diagrams.

We most earnestly ask all classes in London to spend a few moments in considering the facts disclosed by these figures. While the people of London make use of the hospitals in ever-increasing numbers, the figures prove that they are not ready to make an adequate return for the benefits received, but are content to trust to the dead hand to make up any deficiency. Unless this lamentable lassitude in our charity gives place to more earnest endeavour, the ultimate result is not far to seek. In London to-day there are many hospitals unable to take in the patients asking for admission, sometimes because the hospital is not large enough to cope with the district it serves; occasionally because beds are closed for want of funds. Look at this fact from whatever point we will, it is one to make us ashamed. We boast of being citizens of this rich and powerful centre of the greatest Empire the world has known, yet we allow ourselves to remain in imminent danger of the reproach that in spite of our riches, in spite of our power, we grudge the help our suffering and less prosperous brethren need. After all, it is only a very small sacrifice on the part of each individual that is required, and to plead inability is in ninety-nine cases out of a hundred absolute dishonesty. You cannot afford to give a shilling or two to the hospitals, but you never think twice about giving half-a-crown, probably many times a year, for a place in the pit of a theatre. We can all do something. It is our duty: it should be our pleasure. Whether we like it or not, the care of the sick is entrusted to us, and who can be callous enough to neglect such a trust?

### OTHER INSTITUTIONS AND THEIR SPECIAL NEEDS.

**Association for Oral Instruction of the Deaf and Dumb, 11 Fitzroy Square, W.**—Additional support to continue instruction in lip-reading and articulate speech among the deaf and so-called dumb.

**Clergy Orphan Corporation, 33 Parliament Street, S.W.**—Assistance to maintain boys' and girls' schools, and to provide grants from apprenticing fund.

**House of Charity, Greek Street, Soho, W.**—Subscriptions to make good loss in annual income.

**Irish Distressed Ladies Fund, 411 Oxford Street, W.**—Funds to avoid the reduction of pensions and grants to ladies who, owing to non-receipt of rents on property, are in poverty, and are incapacitated by illness or infirmity.

**London Missionary Society, 16 New Bridge Street, E.C.**—Contributions to provide additions to medical staff abroad.

**London Orphan Asylum, Watford. Office, 21 Great St. Helens, E.C.**—Income deficient by £13,500.

**Metropolitan Convalescent Institution, 32 Sackville Street, W.**—Further annual subscriptions and donations to make £13,000 annually.

**Royal Maternity Charity, 31 Finsbury Square, E.C.**—£600 owing to bankers. Loss of income has caused reduction in relief to patients.

**Seaside Home for Sick Women and Children, Whitby.**—£100 increased income. Money to complete redrainage and to pay mortgage.

### OVER TWO MILLION SUFFERERS HELPED BY THE HOSPITALS.

### A SINGLE YEAR'S ROLL-CALL OF THE SICK.

How many patients are annually treated in the London Hospitals? Altogether in the last year, for which complete figures are available, the immense total of *two million one hundred and thirty-seven thousand two hundred and ninety-nine* were treated at the voluntary hospitals and dispensaries of London—together with the endowed hospitals of St. Bartholomew's, Guy's, and St. Thomas's—and the hospitals of the Metropolitan Asylums Board. Of these 761,670 were men, 778,473 women, and 602,156 children.

919,742.
Surgical Patients.

**Patients Suffering from Surgical Diseases.**—Of the whole number of patients received by the hospitals, *nine hundred and nineteen thousand seven hundred and forty-two* required surgical treatment. Let us clearly understand what is meant by "surgical" diseases. They include not only all accidents such as broken bones, fractured skulls, mangled limbs, and all manner of displacements and crushings of sensitive parts and organs, but also abscesses, ulcerations, cancers, and tumours of all kinds; in short all those injuries which may be produced by accident or pathological process, and which may be dealt with either by hand or instrument. That such a vast number of persons in London suffer from one or more of the injuries thus briefly summarised is not easy to realise. They form a very large army indeed, these 919,742 sufferers.

726,341.

Medical Patients.

**Patients Suffering from Medical Diseases.**—*Seven hundred and twenty-six thousand three hundred and forty-one* persons received medical treatment. By medical diseases is meant those diseases which are situated either as to their source and origin or in their entirety in one or the other of the three great cavities of the body. They include rheumatic fever, pneumonia, pleurisy, bronchitis, diseases of the stomach, bowels, liver, kidney, bladder, and pancreas, every kind of heart disease, many forms of brain injury, dyspepsia, constipation, most nervous diseases, and other ailments, many of them serious and many of them dangerous to life, or at least to the useful existence of the individual. Remember that most of these diseases are out of sight, that the diagnosis of their nature and extent, and the successful treatment of them, is dependent on the doctor's scientific knowledge, and then try and realise that in the hospitals of London over 726,000 persons received treatment at the hands of the foremost physicians of the day, free of cost to the patients themselves. Surely when the hospitals plead for help to us who are in health our answer should be a generous one. Good health is a free gift, and since we have received so lavishly we ought certainly, as a thankoffering, to give liberally.

175,374.

Children.

**Patients Treated at Special Hospitals for Children.**—Included in the children mentioned at the commencement of this article are *one hundred and seventy-five thousand three hundred and seventy-four* children who were sent from homes where they could not be properly attended to for treatment in the special hospitals for the little ones. Parents who are happy in the knowledge of the noisy nursery at home, whose very lives echo with the pleasant pattering of little feet, cannot withhold their hands. When the children's hospitals appeal for funds to carry on the work of restoring to health and strength the little ones of this great city,—the children who will one day take our place as the workers in this land of ours,—let the children who are strong and well give to those who are ill and weak.

### THE ROLL-CALL OF THE SICK.—*Continued.*

**Patients Suffering from Eye Affections.** — *One hundred and forty-nine thousand eight hundred and six* persons were treated in the special departments of the general hospitals or by the ophthalmic hospitals of London. It is certain that very many of these cases must have entailed terrible suffering, and many doubtless would have terminated in total loss of sight but for the skilful treatment they have received at the hospitals. Who can say how many have been saved from becoming practically helpless in the world?

149,806. Eye.

**Diseases of Women and Motherhood.**—*One hundred and thirteen thousand four hundred and fifty-nine* women were treated at the metropolitan voluntary hospitals for those diseases which are peculiar to their sex. Here it is not only our sympathy which is appealed to, but our patriotism as well. Here there is an actual demand for the payment of a debt we most justly owe. The very heart and strength of the nation lies in the home life, and the soul of the home life is the woman—the mother. The majority of us are to-day what we are because of the influences brought to bear upon us in the home.

113,459. Women.

**Patients Suffering from Diseases of the Ear, Nose, and Throat.**—At the special hospitals or special departments devoted to these diseases *seventy five thousand nine hundred and sixty-nine* were treated. The affections and diseases of these organs, which are intimately connected, involve temporary and often permanent impairment of hearing, swallowing, and breathing. These functions are performed with so little effort on our part that, unless experience has taught us, it is difficult to understand what it would mean to us if we suddenly had to suffer from one or other of these affections.

75,969. Ear and Throat.

**Patients Suffering from Diseases of the Skin.**—During the year *fifty eight thousand and forty-three* persons were treated for skin diseases in London. It is perhaps, more difficult to bring home to people the claim which sufferers from skin diseases have upon their sympathy than it is in any of the other classes of disease which we are considering. There is not, as a rule, the pain, nor the danger to life, nor even such risk of permanent disablement as is the case with many of the others ; but let us remember what the result would be were there no hospitals for the sufferers to go to.

58,043. Skin.

**Patients Suffering from Consumption.**—*Forty-nine thousand three hundred and twenty-six* patients suffering from phthisis or consumption were treated at the consumption hospitals of London during the year. The very word consumption makes us afraid. There are few of us who have not seen something of its ravages, of its cruelty. Truly may consumption be called the curse of our climate. It respects neither persons nor estate, neither rich nor poor, the old or the young.

49,326. Consumption.

**Patients Suffering from Fever.**—The number of persons who suffered from various forms of fever during the year was 23,035. This figure is, however, a misleading one, because the term fever includes much besides the class of fevers which are usually removed to these hospitals. Measles, for instance, prevails in London to such an extent that more deaths occur from it than from scarlet fever. The excellent service rendered by the London Fever Hospital entitles it to the gratitude of all householders.

23,035. Fever.

**Patients Suffering from Paralysis and Diseases of the Nerves.**—*Twenty-one thousand five hundred and seventy-eight* stricken with paralysis and similar diseases of the brain received treatment at the general hospitals and those hospitals devoted to these maladies. To workers busy with hand and brain these sufferers must particularly appeal. It is impossible to dissociate nervous breakdown from the toil and hurry of existence, especially in a vast centre like London. It is appalling to think that at any moment any one of us may be struck down, perhaps without the slightest warning. No disease is more sudden than paralysis, surely none more pitiful.

21,578. Paralysis.

So this great army of sufferers, numbering nearly two and a quarter millions, claims our sympathy and our help year by year. To the strong, to those in health who are able to provide for those dependent upon them, to those who know what ill-health means, who have suffered from disease of one kind or another, and who, either in the hospital or under the skill and care of the doctors and nurses trained in the hospital, have been restored to health and usefulness, we confidently appeal on behalf of the London hospitals.

### THE ROLL-CALL OF THE SICK.

| | | | |
|---|---|---|---|
| Sufferers needing Surgical Aid | 919,742 | Sufferers from Skin Diseases | 58,043 |
| Sufferers needing Medical Care | 726,341 | Consumptives | 49,326 |
| Sufferers from Eye Troubles | 149,806 | Fever Patients | 23,035 |
| Diseases of Women | 113,459 | Paralysis | 21,578 |
| Diseases of the Ear, Nose, and Throat | 75,969 | Total | 2,137,299 |

### 1904.

### A Year's Work in the Hospitals and Medical Charities of London.

### NEWINGTON AND SOUTH DISTRICT.

Comprising Battersea, Wandsworth, Tooting, Balham, Streatham, Brixton, Lambeth, Newington, Southwark, Bermondsey, Camberwell, Greenwich, Deptford, Lewisham, Blackheath, Woolwich, &c.

| No. of Beds. | No. of Beds Daily Occupied. | Hospitals. | In-patients. | Out-patients. | Total Expenditure. | Income. Charitable. | Income. Proprietary. | Income. Patients' Payments. | Total Income. | Legacies not included in previous column. |
|---|---|---|---|---|---|---|---|---|---|---|
| | | | | | £ | £ | £ | £ | £ | £ |
| 602 | 515 | Guy's ... ... ... ... | 8,070 | 137,498 | 73,159 | 18,072 | 34,580 | 4,511 | 57,163 | 4,857 |
| 11 | 7 | Phillips' Memorial Homœopathic | 172 | 809 | 896 | 484 | 147 | 285 | 916 | ... |
| 25 | 21 | Miller ... ... ... ... | 356 | 19,797 | 3,723 | 3,216 | 597 | ... | 3,813 | 2,485 |
| 41 | 30 | St. John's, Lewisham ... ... | 246 | ... | 2,790 | 1,671 | 51 | 589 | 2,311 | ... |
| 269 | 232 | Seamen's ... ... ... ... | 2,299 | 23,753 | 24,586 | 10,838 | 4,004 | 1,499 | 16,341 | 1,597 |
| 76 | 62 | Evelina, for Children ... ... | 1,078 | 34,131 | 7,511 | 4,643 | 3,571 | 127 | 8,341 | ... |
| 57 | 41 | Home for Sick Children... ... | 222 | 1,718 | 2,849 | 1,180 | 273 | 347 | 1,800 | 50 |
| 24 | 22 | General Lying-In... ... ... | 525 | 1,906 | 4,887 | 1,061 | 3,735 | ... | 4,796 | 91 |
| 40 | 17 | Clapham Maternity & Dispensary | 383 | 6,237 | 2,107 | 400 | 667 | 837 | 1,904 | ... |
| Rebui | lding | Royal, Waterloo ... ... ... | 149 | 5,275 | 3,186 | 2,672 | 930 | 88 | 3,690 | 2,100 |
| 42 | 32 | Royal Eye ... ... ... ... | 652 | 21,580 | 3,601 | 2,195 | 213 | 560 | 2,968 | 1,520 |
| 32 | 15 | Beckenham Cottage ... ... | 246 | 528 | 1,255 | 1,080 | ... | 151 | 1,231 | ... |
| 20 | 16 | Blackheath Cottage ... ... | 201 | 1,123 | 1,880 | 959 | 34 | 324 | 1,317 | 100 |
| 32 | 25 | Bromley Cottage ... ... ... | 441 | ... | 1,871 | 1,533 | 257 | 154 | 1,944 | 50 |
| 22 | 12 | Chislehurst, &c., Cottage ... | 210 | ... | 986 | 744 | 1 | 333 | 1,078 | ... |
| 14 | 10 | Eltham Cottage ... ... ... | 160 | ... | 757 | 755 | 42 | 202 | 999 | 25 |
| 14 | 7 | Sidcup Cottage ... ... ... | 145 | 62 | 612 | 384 | 24 | 158 | 566 | ... |
| 18 | 13 | Livingstone Cottage ... ... | 140 | ... | 1,043 | 1,134 | 15 | 40 | 1,189 | ... |
| 10 | 8 | Woolwich and Plumstead Cottage | 90 | 38 | 610 | 602 | 10 | 92 | 704 | ... |
| 30 | 27 | Bolingbroke Hospital ... ... | 447 | 6,694 | 3,900 | 2,721 | 207 | 571 | 3,499 | 5 350 |
| 14 | 10 | Victoria Hospital, Kingston ... | 152 | ... | 938 | 859 | 57 | 101 | 1,017 | ... |
| 1,393 | 1,122 | | 16,384 | 261,149 | 143,147 | 57,203 | 49,415 | 10,969 | 117,587 | 18,225 |
| | | **Dispensaries.** | | | | | | | | |
| ... | ... | Battersea Provident ... ... | ... | 30,074 | 3,943 | 117 | 57 | 3,534 | 3,708 | ... |
| ... | ... | Blackfriars, Provident ... ... | ... | 1,776 | 247 | 103 | ... | 146 | 249 | ... |
| ... | ... | Brixton, &c. ... ... ... | ... | 5,786 | 674 | 514 | 42 | 122 | 678 | ... |
| ... | ... | Camberwell Provident ... ... | ... | 7,655 | 1,778 | 312 | 198 | 1,285 | 1,795 | ... |
| ... | ... | Clapham ... ... ... ... | ... | 1,460 | 339 | 297 | 32 | 121 | 450 | ... |
| ... | ... | Deptford Medical Mission ... | ... | 2,983 | 437 | 168 | 31 | 74 | 273 | ... |
| ... | ... | East Dulwich Provident ... | ... | 6,851 | 943 | 124 | 14 | 924 | 1,062 | ... |
| ... | ... | Forest Hill Provident ... .. | ... | 2,849 | 673 | 218 | 5 | 445 | 668 | ... |
| ... | ... | Greenwich Provident ... ... | ... | 2,920 | 665 | 68 | 18 | 578 | 664 | ... |
| ... | ... | Royal South London ... ... | ... | 3,375 | 623 | 563 | 56 | ... | 619 | ... |
| ... | ... | South Lambeth, &c. ... ... | ... | 1,751 | 485 | 479 | 6 | 134 | 619 | ... |
| ... | | Walworth Provident ... ... | ... | 803 | 270 | 81 | 29 | 162 | 272 | ... |
| ... | ... | Wandsworth Common ... ... | ... | 1,493 | 290 | 44 | ... | 247 | 291 | ... |
| ... | ... | Woolwich, &c., Provident ... | ... | 3,565 | 498 | 46 | 1 | 418 | 465 | ... |
| 1,393 | 1,122 | | 16,384 | 334,490 | 155,012 | 60,337 | 49,904 | 19,159 | 129,400 | 18,225 |

### WESTMINSTER DISTRICT.—Comprising Westminster City and Liberties.

| No. of Beds. | No. of Beds Daily Occupied. | Hospitals. | In-patients. | Out-patients. | Total Expenditure. | Income. Charitable. | Income. Proprietary. | Income. Patients' Payments. | Total Income. | Legacies not included in previous column. |
|---|---|---|---|---|---|---|---|---|---|---|
| | | | | | £ | £ | £ | £ | £ | £ |
| 164 | 150 | Charing Cross ... ... ... | 2,184 | 19,138 | 48,460 | 9,703 | 2,386 | ... | 12,089 | 2,663 |
| 224 | 185 | King's College ... ... ... | 2,609 | 18,619 | 23,039 | 11,095 | 4,610 | 21 | 15,726 | 2,671 |
| 213 | 173 | Westminster ... ... ... | 2,512 | 23,420 | 20,690 | 8,180 | 3,789 | ... | 11,969 | 557 |
| 155 | 147 | Ventnor, for Consumption ... | 817 | ... | 15,268 | 5,184 | 2,227 | 4,256 | 11,667 | 640 |
| 24 | 15 | Grosvenor, for Women & Children | 190 | 4,297 | 2,373 | 1,608 | 105 | 505 | 2,218 | ... |
| 60 | 56 | Hospital for Women ... ... | 838 | 4,090 | 6,651 | 5,115 | 226 | 1,156 | 6,497 | 2,560 |
| 26 | 21 | Gordon, for Fistula ... ... | 336 | 969 | 2,183 | 739 | 55 | 1,427 | 2,221 | ... |
| 25 | 24 | National, for Diseases of Heart... | 192 | 2,934 | 2,755 | 1,344 | 93 | 676 | 2,113 | 300 |
| 40 | 29 | Royal Westminster Ophthalmic... | 745 | 10,158 | 2,452 | 2,093 | 746 | ... | 2,839 | 900 |
| ... | ... | Royal Orthopædic ... ... | ... | 687 | 1,841 | 917 | 389 | 115 | 1,421 | ... |
| 9 | 6 | Royal Ear ... ... ... ... | 177 | 1,906 | 860 | 424 | ... | 430 | 854 | ... |
| ... | ... | Royal Dental ... ... ... | ... | 28,836 | 3,131 | 2,992 | 70 | 142 | 3,204 | 2,600 |
| 26 | 24 | St. Peter's, for Stone ... ... | 449 | 4,074 | 2,553 | 1,151 | 521 | 2,279 | 3,951 | 1,700 |
| 45 | 32 | St. John's, for Skin ... ... | 239 | 7,404 | 4,573 | 1,917 | 28 | 2,682 | 4,627 | ... |
| 33 | 29 | Hospital for Diseases of Throat... | 845 | 11,235 | 4,899 | 1,196 | 47 | 4,125 | 5,368 | 50 |
| 1,044 | 891 | | 12,133 | 137,767 | 141,728 | 53,658 | 15,292 | 17,814 | 86,764 | 14,641 |
| | | **Dispensaries.** | | | | | | | | |
| ... | ... | Public ... ... ... ... | ... | 1,745 | 668 | 371 | 224 | ... | 595 | ... |
| ... | ... | St. George and St. James ... | ... | 3,649 | 562 | 548 | 1 | 24 | 573 | ... |
| ... | ... | St. George's, Hanover Square ... | ... | 1,710 | 507 | 365 | ... | 148 | 513 | ... |
| ... | ... | Western ... ... ... ... | ... | 10,814 | 1,551 | 305 | 454 | 768 | 1,527 | ... |
| ... | ... | Westminster General ... ... | ... | 6,219 | 731 | 465 | 188 | 93 | 746 | ... |
| 1,044 | 891 | | 12,133 | 161,904 | 145,747 | 55,712 | 16,159 | 18,847 | 90,718 | 14,641 |

### ST MARYLEBONE AND WEST CENTRAL DISTRICT.

Comprising St. Marylebone, St. John's Wood, Bloomsbury, Holborn, &c.

| No. of Beds. | No. of Beds Daily Occupied. | HOSPITALS. | In-patients. | Out-patients. | Total Expenditure. | Income. | | | Total Income. | Legacies not included in previous column. |
|---|---|---|---|---|---|---|---|---|---|---|
| | | | | | | Charitable. | Proprietary. | Patients' Payments. | | |
| | | | | | £ | £ | £ | £ | £ | £ |
| 70 | 52 | French ... ... ... ... | 833 | 5,559 | 4,391 | 4,500 | 14 | ... | 4,514 | 1,982 |
| 50 | 26 | Italian ... ... ... ... | 538 | 11,761 | 2,691 | 2,694 | 291 | ... | 2,985 | ... |
| 103 | 75 | London Homœopathic ... ... | 1,016 | 23,488 | 10,286 | 3,010 | 2,674 | 984 | 6,668 | 600 |
| 110 | 63 | SS. John and Elizabeth ... ... | 249 | ... | 4,971 | 6,228 | 471 | 696 | 7,395 | 525 |
| 402 | 373 | The Middlesex ... ... ... | 5,047 | 48,707 | 47,964 | 20,492 | 9,310 | ... | 29,802 | 12,638 |
| 97 | 89 | Alexandra, for Children... ... | 173 | 328 | 6,331 | 4,938 | 398 | 554 | 5,890 | 450 |
| 252 | 191 | Hospital for Sick Children ... | 2,537 | 37,617 | 20,454 | 8,074 | 5,223 | ... | 13,297 | 4,808 |
| 26 | 21 | S. Monica's, for Children ... | 105 | ... | 1,339 | 770 | 114 | 262 | 1,146 | 750 |
| 30 | 18 | British Lying-In ... ... ... | 449 | 618 | 2,927 | 578 | 1,916 | 86 | 2,580 | ... |
| 74 | 61 | Queen Charlotte's Lying-In ... | 1,456 | 1,754 | 6,233 | 4,763 | 597 | 111 | 5,471 | 10,243 |
| 52 | 38 | New Hospital for Women ... | 539 | 13,513 | 5,839 | 2,419 | 493 | 1,621 | 4,533 | 3,618 |
| 47 | 44 | Samaritan Free ... ... ... | 565 | 7,042 | 6,069 | 3,205 | 296 | ... | 3,501 | 5,400 |
| 200 | 184 | National for the Paralysed, &c.... | 1,113 | 6,574 | 18,992 | 9,293 | 1,770 | 2,328 | 13,391 | 4,304 |
| 38 | 27 | Hospital for Epilepsy, &c. ... | 130 | 881 | 2,488 | 1,341 | 109 | 712 | 2,162 | 2,261 |
| 50 | 36 | West End, for Epilepsy, &c. ... | 342 | 4,824 | 6,388 | 4,463 | 236 | 495 | 5,194 | ... |
| 26 | 19 | Central London Ophthalmic ... | 369 | 12,820 | 1,871 | 1,799 | 44 | ... | 1,843 | ... |
| 12 | 9 | Western Ophthalmic ... ... | 295 | 14,062 | 1,123 | 920 | 185 | 7 | 1,112 | 900 |
| 60 | 52 | National Orthopædic ... ... | 270 | 1,684 | 2,473 | 1,833 | 55 | 1,170 | 3,058 | ... |
| 20 | 15 | Hospital for Gentlewomen ... | 198 | ... | 2,646 | 1,303 | 204 | 1,015 | 2,522 | 50 |
| ... | ... | National Dental ... ... ... | ... | 19,145 | 1,792 | 298 | ... | 1,209 | 1,507 | ... |
| 16 | 10 | London Throat ... ... ... | 675 | 4,740 | 1,609 | 482 | ... | 1,112 | 1,594 | ... |
| 33 | 20 | Oxygen Hospital... ... ... | 66 | ... | 1,222 | 465 | 19 | 724 | 1,208 | ... |
| ... | ... | Western Skin ... ... ... | ... | 1,099 | 340 | 277 | ... | 100 | 377 | ... |
| 1,768 | 1,423 | | 16,965 | 216,216 | 160,439 | 84,145 | 24,419 | 13,186 | 121,750 | 48,529 |
| | | DISPENSARIES. | | | | | | | | |
| ... | ... | Bloomsbury Provident ... ... | ... | 1,662 | 243 | 62 | ... | 183 | 245 | ... |
| ... | ... | London Medical Mission ... | ... | 9,657 | 1,584 | 1,056 | 53 | 284 | 1,393 | 53 |
| ... | ... | Margaret Street, for Consumption | ... | 936 | 503 | 306 | ... | 164 | 470 | ... |
| ... | ... | Portland Town ... ... ... | ... | 1,447 | 163 | 147 | 4 | 12 | 163 | ... |
| ... | ... | St. John's Wood Provident ... | ... | 6,632 | 720 | 287 | 17 | 406 | 710 | ... |
| ... | ... | St. Marylebone General... ... | ... | 3,597 | 865 | 463 | 164 | 161 | 788 | ... |
| ... | ... | Western General... ... ... | ... | 15,360 | 1,304 | 856 | 66 | 54 | 976 | ... |
| 1,768 | 1,423 | | 16,965 | 255,507 | 165,821 | 87,322 | 24,723 | 14,450 | 126,495 | 48,582 |

### STRATFORD AND EAST-END DISTRICT.

Comprising Bethnal Green, Tower Hamlets, West Ham, Whitechapel, Hackney, Stepney, Limehouse, Poplar, and the East.

| No. of Beds. | No. of Beds Daily Occupied. | HOSPITALS. | In-patients. | Out-patients. | Total Expenditure. | Income. | | | Total Income. | Legacies not included in previous column. |
|---|---|---|---|---|---|---|---|---|---|---|
| | | | | | | Charitable. | Proprietary. | Patients' Payments. | | |
| | | | | | £ | £ | £ | £ | £ | £ |
| 130 | 121 | German ... ... ... ... | 1,637 | 31,612 | 10,414 | 7,678 | 2,906 | 466 | 11,050 | 420 |
| 816 | 705 | London ... ... ... ... | 12,863 | 206,386 | 188,067 | 56,300 | 26,498 | 2,242 | 85,040 | 12,533 |
| 50 | 32 | Mildmay Mission Hospital ... | 583 | 12,059 | 4,452 | 3,304 | 666 | 149 | 4,119 | ... |
| 103 | 74 | Poplar ... ... ... ... | 1,349 | 11,233 | 9,918 | 9,543 | 1,132 | 201 | 10,876 | 1,470 |
| 60 | 50 | West Ham, &c. ... ... ... | 707 | 23,974 | 5,991 | 6,160 | 489 | ... | 6,649 | 270 |
| 45 | 27 | Walthamstow, &c. ... ... | 554 | 3,592 | 2,013 | 1,215 | 200 | ... | 1,415 | ... |
| 164 | 124 | City of London for Dis. of the Chest | 833 | 9,772 | 12,501 | 9,882 | 304 | ... | 10,186 | 5,378 |
| 135 | 120 | East London for Children ... | 2,053 | 35,344 | 10,761 | 6,915 | 1,245 | ... | 8,160 | 1,387 |
| 35 | 29 | St. Mary's, Plaistow, for Children | 659 | 18,280 | 3,523 | 3,351 | 159 | 178 | 3,688 | 50 |
| 23 | 14 | East End Mothers' Home ... | 377 | 453 | 2,026 | 1,614 | 781 | 67 | 2,462 | ... |
| 13 | 10 | Canning Town Cottage ... ... | 128 | 3,438 | 1,042 | 576 | 220 | 199 | 995 | ... |
| 14 | 8 | Passmore Edwards Cottage, T'lb'ry | 104 | 513 | 892 | 903 | 55 | 2 | 960 | ... |
| 20 | 12 | East Ham Cottage ... ... | 249 | 2,866 | 1,404 | 809 | 76 | 6 | 891 | ... |
| 1,608 | 1,326 | | 22,096 | 359,522 | 253,004 | 108,250 | 34,731 | 3,510 | 146,491 | 21,508 |
| | | DISPENSARIES. | | | | | | | | |
| ... | ... | All Saints, Buxton Street ... | ... | 1,376 | 377 | 434 | ... | ... | 434 | ... |
| ... | ... | Eastern ... ... ... ... | ... | 8,028 | 990 | 181 | 294 | 291 | 766 | ... |
| ... | ... | Hackney Provident ... ... | ... | 1,085 | 303 | 91 | ... | 220 | 311 | ... |
| ... | ... | London ... ... ... ... | ... | 2,649 | 485 | 127 | 261 | ... | 388 | 250 |
| ... | ... | Queen Adelaide's... ... ... | ... | 6,477 | 624 | 522 | 225 | ... | 747 | 52 |
| ... | ... | Tower Hamlets ... ... ... | ... | 3,550 | 670 | 667 | 27 | 146 | 840 | ... |
| ... | ... | Whitechapel Provident ... ... | ... | 3,765 | 765 | 120 | ... | 629 | 749 | ... |
| 1,608 | 1,326 | | 22,096 | 386,452 | 257,218 | 110,392 | 35,538 | 4,796 | 150,726 | 21,810 |

### ISLINGTON AND NORTH-WEST DISTRICT.

Comprising Islington, Holloway, Highbury, Hampstead, Highgate, St. Pancras, Stoke Newington, Tottenham, &c.

| No. of Beds. | No. of Beds Daily Occupied. | HOSPITALS. | In-patients. | Out-patients. | Total Expenditure. | Income. | | | Total Income. | Legacies not included in previous column. |
|---|---|---|---|---|---|---|---|---|---|---|
| | | | | | | Charitable. | Proprietary. | Patients' Payments. | | |
| | | | | | £ | £ | £ | £ | £ | £ |
| 157 | 145 | Great Northern Central ... ... | 1,982 | 27,742 | 15,318 | 6,755 | 1,245 | 718 | 8,718 | 3,725 |
| 35 | 28 | Hampstead General Hospital ... | 432 | 2,538 | 3,451 | 3,358 | 7 | 364 | 3,729 | |
| 117 | 86 | London Temperance ... ... | 1,337 | 24,721 | 10,342 | 4,072 | 1,821 | 328 | 6,221 | 2,915 |
| 50 | 45 | North-West London ... ... | 609 | 24,251 | 4,856 | 6,626 | 166 | 138 | 6,930 | 150 |
| 73 | 70 | Tottenham ... ... ... | 1,093 | 20,902 | 6,597 | 8,883 | 195 | ... | 9,078 | ... |
| 191 | 166 | University College ... ... | 2,744 | 58,321 | 26,778 | 11,969 | 4,044 | 76 | 16,089 | 5,743 |
| 245 | 142 | Mount Vernon for Consumption | 696 | 5,243 | 11,651 | 8,685 | 112 | ... | 8,797 | 2,800 |
| 14 | 12 | Children's Home Hospital, Barnet | 55 | ... | 565 | 470 | ... | 84 | 554 | ... |
| 200 | 45 | London Fever ... ... ... | 446 | ... | 11,833 | 6,785 | 1,994 | 1,252 | 10,031 | 3,324 |
| 28 | 16 | Invalid Asylum ... ... ... | 202 | ... | 1,108 | 483 | 255 | 124 | 862 | ... |
| 15 | 12 | Enfield Cottage ... ... ... | 177 | ... | 574 | 739 | 33 | 12 | 784 | ... |
| 27 | 17 | Memorial Cottage, Mildmay ... | 138 | ... | 1,866 | 336 | 984 | 48 | 1,368 | ... |
| 27 | 14 | St. Saviour's Hospital ... ... | 137 | ... | 2,113 | 1,130 | 3 | 882 | 2,015 | 20 |
| 48 | 39 | Friedenheim Hospital ... ... | 149 | ... | 3,774 | 2,340 | 120 | 197 | 2,657 | 500 |
| 25 | 19 | Willesden Cottage ... ... | 220 | 212 | 1,394 | 1,092 | 113 | 97 | 1,302 | ... |
| 30 | 7 | Bushey Heath Cottage ... ... | 102 | ... | 719 | 734 | 123 | 197 | 1,054 | 100 |
| 20 | 19 | Santa Claus Home ... ... | 33 | ... | 963 | 858 | 21 | 33 | 912 | ... |
| 36 | 24 | Hospital for Incurable Children | 37 | ... | 1,378 | 503 | 86 | 352 | 941 | ... |
| 1,338 | 906 | | 10,589 | 163,930 | 105,280 | 65,818 | 11,322 | 4,902 | 82,042 | 19,277 |
| | | DISPENSARIES. | | | | | | | | |
| ... | ... | Camden Provident ... ... | ... | 1,047 | 321 | 34 | 45 | 236 | 315 | ... |
| ... | ... | Childs' Hill, Provident ... ... | ... | 2,605 | 333 | 45 | 13 | 276 | 334 | ... |
| ... | ... | Hampstead Provident ... ... | ... | 11,531 | 1,109 | 328 | 12 | 781 | 1,121 | ... |
| ... | ... | Holloway and North Islington ... | ... | 2,337 | 697 | 291 | 40 | 203 | 534 | ... |
| ... | ... | Islington ... ... ... ... | ... | 10,327 | 931 | 393 | 16 | 604 | 1,013 | 100 |
| ... | ... | St. Pancras and Northern ... | ... | 3,321 | 572 | 193 | 161 | 115 | 469 | ... |
| ... | ... | St. Pancras Medical Mission ... | ... | 1,690 | 246 | 230 | 18 | 20 | 268 | ... |
| ... | ... | Stamford Hill, &c. ... ... | ... | 7,478 | 774 | 679 | 167 | ... | 846 | 100 |
| ... | ... | Islington Medical Mission ... | ... | 2,926 | 507 | 409 | 28 | 53 | 490 | ... |
| ... | ... | Kentish Town Medical Mission | ... | 1,038 | 226 | 197 | 5 | 17 | 219 | ... |
| 1,338 | 906 | | 10,589 | 208,230 | 110,996 | 68,617 | 11,827 | 7,207 | 87,651 | 19,477 |

### KENSINGTON AND WEST DISTRICT.

Comprising Kensington, Paddington, Bayswater, Kilburn, Chelsea, Brompton, Fulham, Hammersmith, Chiswick, Brentford, Acton, Ealing, &c.

| | | | | | | | | | | |
|---|---|---|---|---|---|---|---|---|---|---|
| | | HOSPITALS. | | | £ | £ | £ | £ | £ | £ |
| 18 | 14 | Queen's Jubilee ... ... ... | 282 | 13,664 | 2,219 | 1,403 | 39 | ... | 1,442 | ... |
| 351 | 303 | St. George's ... ... ... | 4,530 | 38,925 | 42,972 | 12,599 | 14,634 | ... | 27,233 | 6,290 |
| 281 | 249 | St. Mary's ... ... ... ... | 3,989 | 45,513 | 31,564 | 11,548 | 2,867 | 223 | 14,638 | 7,006 |
| 138 | 130 | West London ... ... ... | 2,101 | 38,528 | 15,314 | 9,987 | 539 | ... | 10,526 | 1,320 |
| 318 | 303 | Hospital for Consumption ... | 1,404 | 12,447 | 34,666 | 14,170 | 6,025 | ... | 20,195 | 12,208 |
| 30 | 17 | Belgrave, for Children ... ... | 397 | 9,579 | 2,542 | 2,078 | 89 | ... | 2,167 | ... |
| 50 | 50 | Cheyne, for Sick & Incurable Chldn | 20 | ... | 2,708 | 1,736 | 610 | 282 | 2,628 | 900 |
| 12 | 8 | Kensington, for Children .. | 163 | 4,982 | 1,378 | 957 | 123 | 6 | 1,086 | 215 |
| 46 | 37 | Paddington Green, for Children | 763 | 16,392 | 4,944 | 3,477 | 253 | 387 | 4,117 | 2,700 |
| 154 | 93 | Victoria, for Children ... ... | 1,295 | 20,715 | 8,054 | 5,653 | 651 | 425 | 6,729 | 2,435 |
| 50 | 43 | Chelsea, for Women ... ... | 755 | 3,565 | 5,868 | 3,274 | 248 | 960 | 4,482 | 1,520 |
| 105 | 86 | Cancer ... ... ... ... | 721 | 1,763 | 13,224 | 6,154 | 4,434 | ... | 10,588 | 9,725 |
| 135 | 92 | Female Lock ... ... ... | 568 | ... | 5,122 | 2,623 | 31 | 1,452 | 4,106 | 1,170 |
| 20 | 19 | Banstead Surgical Home ... | 121 | ... | 570 | 253 | 11 | 322 | 586 | ... |
| 16 | 9 | Acton Cottage ... ... ... | 154 | 895 | 1,828 | 1,383 | 19 | 56 | 1,458 | 676 |
| 16 | 7 | Epsom and Ewell Cottage ... | 105 | ... | 752 | 633 | 29 | 155 | 817 | ... |
| 16 | 11 | Hounslow Cottage ... ... | 366 | 835 | 703 | 433 | 183 | 71 | 687 | 50 |
| 31 | 19 | Reigate and Redhill Cottage ... | 298 | ... | 1,284 | 1,027 | 18 | 202 | 1,247 | 200 |
| 16 | 8 | Wimbledon Cottage ... ... | 152 | ... | 694 | 484 | 27 | 132 | 643 | 300 |
| 12 | 7 | Wimbledon, South, Cottage ... | 153 | ... | 809 | 616 | 2 | 117 | 735 | ... |
| 1,815 | 1,505 | | 18,338 | 207,803 | 177,215 | 80,488 | 30,832 | 4,790 | 116,110 | 46,715 |
| | | DISPENSARIES. | | | | | | | | |
| ... | ... | Brompton Provident ... ... | ... | 1,568 | 366 | 84 | 76 | 195 | 355 | ... |
| ... | ... | Chelsea, &c. ... ... ... | ... | 2,774 | 1,075 | 263 | 106 | 82 | 451 | 100 |
| ... | ... | Chelsea Provident ... ... | ... | 595 | 249 | 60 | 17 | 170 | 247 | ... |
| ... | ... | Kensal Town Provident ... ... | ... | 859 | 299 | 51 | ... | 249 | 300 | ... |
| ... | ... | Kilburn, Maida Vale ... ... | ... | 1,518 | 457 | 371 | 50 | ... | 421 | ... |
| ... | ... | Kilburn Provident ... ... | ... | 7,800 | 1,346 | 71 | 9 | 1,289 | 1,369 | ... |
| ... | ... | Notting Hill Provident ... ... | ... | 837 | 281 | 87 | ... | 181 | 268 | ... |
| ... | ... | Paddington Provident ... ... | ... | 2,862 | 615 | 183 | 23 | 329 | 535 | ... |
| ... | ... | Royal Pimlico Provident... ... | ... | 3,307 | 819 | 321 | 111 | 388 | 820 | ... |
| ... | ... | Westbourne Provident ... ... | ... | 1,181 | 407 | 70 | 51 | 338 | 459 | ... |
| 1,815 | 1,505 | | 18,338 | 231,104 | 183,129 | 82,049 | 31,275 | 8,011 | 121,335 | 46,815 |

### CITY AND EAST CENTRAL DISTRICT.

Comprising the City, St. Luke's, Shoreditch, Finsbury, and Clerkenwell.

| No. of Beds. | No. of Beds Daily Occupied. | HOSPITALS. | In-patients. | Out-patients. | Total Expenditure. | Income. | | | Total Income. | Legacies not included in previous column. |
|---|---|---|---|---|---|---|---|---|---|---|
| | | | | | | Charitable. | Proprietary. | Patients' Payments. | | |
| | | | | | £ | £ | £ | £ | £ | £ |
| 111 | 96 | Metropolitan ... ... ... | 1,220 | 36,167 | 15,159 | 10,689 | 531 | 7 | 11,227 | 4,508 |
| 165 | 124 | Royal Free ... ... ... | 2,216 | 42,050 | 14,206 | 5,710 | 1,514 | ... | 7,224 | 9,185 |
| 80 | 58 | Royal, for Diseases of the Chest. | 715 | 6,869 | 7,438 | 4,644 | 284 | ... | 4,928 | 7,024 |
| 114 | 95 | North-Eastern, for Children ... | 1,258 | 24,445 | 11,477 | 11,719 | 219 | 617 | 12,555 | ... |
| 36 | 24 | City of London Lying-In ... | 601 | 2,533 | 7,195 | 813 | 4,135 | ... | 4,948 | ... |
| 40 | 34 | St. Mark's, for Fistula ... ... | 477 | 1,813 | 4,046 | 2,577 | 632 | 269 | 3,478 | 829 |
| 138 | 102 | Royal London Ophthalmic ... | 2,194 | 43,038 | 12,198 | 11,114 | 371 | ... | 11,485 | 1,686 |
| 45 | 28 | City Orthopædic ... ... ... | 228 | 1,301 | 3,337 | 1,965 | 4 | ... | 1,969 | 250 |
| 16 | 12 | Central London Throat and Ear. | 365 | 9,751 | 2,735 | 922 | ... | 1,914 | 2,836 | ... |
| 745 | 573 | | 9,274 | 167,967 | 77,791 | 50,153 | 7,690 | 2,807 | 60,650 | 23,482 |
| | | DISPENSARIES. | | | | | | | | |
| ... | ... | Billingsgate Medical Mission ... | ... | 4,156 | 998 | 811 | ... | 79 | 890 | ... |
| ... | ... | City ... ... ... ... ... | ... | 5,834 | 947 | 788 | 88 | ... | 876 | ... |
| ... | ... | City of London and East London | ... | 27,581 | 1,846 | 114 | 155 | 2,307 | 2,576 | ... |
| ... | ... | Farringdon General ... ... | ... | 2,497 | 602 | 335 | ... | 207 | 542 | .. |
| ... | ... | Finsbury ... ... ... ... | ... | 9,574 | 1,031 | 592 | 192 | 231 | 1,015 | ... |
| ... | ... | Metropolitan ... ... ... | ... | 11,136 | 888 | 378 | 112 | 246 | 736 | ... |
| ... | ... | Royal General ... ... ... | ... | 2,751 | 711 | 259 | 349 | 86 | 694 | ... |
| 745 | 573 | | 9,274 | 231,496 | 84,814 | 53,430 | 8,586 | 5 963 | 67,979 | 23,482 |

### THE MEDICAL CHARITIES OF LONDON.—A SUMMARY OF THE WORK DONE IN 1904.

It will be seen from the following summary that the Voluntary Hospitals and Medical Charities of London, during the twelve months ending December 31st, 1904, relieved over One million nine hundred and fourteen thousand patients at a cost of £1,102,737. The Ordinary Income only amounted to £774,304, leaving a deficiency of £328,433 on the year's work. The legacies received in 1904 amounted to £193,032, being £88,657 less than the amount received in 1903.

| No. of Beds. | No. of Beds Daily Occupied. | HOSPITALS AND DISPENSARIES. | In-patients. | Out-patients. | Total Expenditure. | Income. | | | Total Income. | Legacies not included in previous column. |
|---|---|---|---|---|---|---|---|---|---|---|
| | | | | | | Charitable. | Proprietary. | Patients' Payments. | | |
| | | | | | £ | £ | £ | £ | £ | £ |
| 1,393 | 1,122 | Newington and South District ... | 16,384 | 334,490 | 155,012 | 60,337 | 49,904 | 19,159 | 129,400 | 18,225 |
| 745 | 573 | City and East Central District... | 9,274 | 231,496 | 84,814 | 53,430 | 8,586 | 5,963 | 67,979 | 23,482 |
| 1,044 | 891 | Westminster District ... ... | 12,133 | 161,904 | 145,747 | 55,712 | 16,159 | 18,847 | 90,718 | 14,641 |
| 1,768 | 1,423 | St. Marylebone and West Central District ... ... ... | 16,965 | 255,507 | 165,821 | 87,322 | 24,723 | 14,450 | 126,495 | 48,582 |
| 1,815 | 1,505 | Kensington and West District ... | 18,338 | 231,104 | 183,129 | 82,049 | 31,275 | 8,011 | 121,335 | 46,815 |
| 1,338 | 906 | Islington & North-West District | 10,589 | 208,230 | 110,996 | 68,617 | 11,827 | 7,207 | 87,651 | 19,477 |
| 1,608 | 1,326 | Stratford and East-End District | 22,096 | 386,452 | 257,218 | 110,392 | 35,538 | 4,796 | 150,726 | 21,810 |
| 9,711 | 7,746 | | 105,779 | 1,809,183 | 1,102,737 | 517,859 | 178,012 | 78,433 | 774,304 | 193,032 |

## Figures and Tables

**Figure f1:**
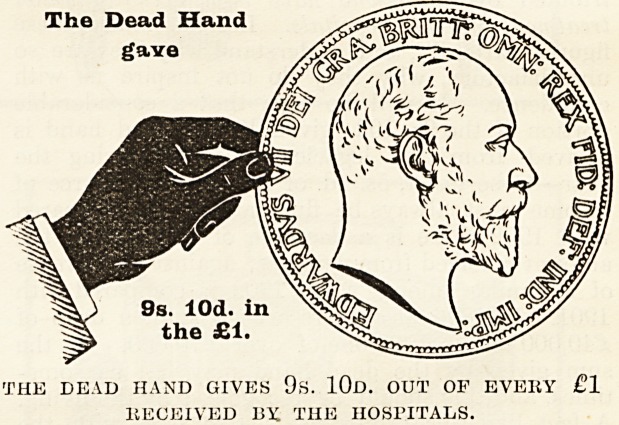


**Figure f2:**
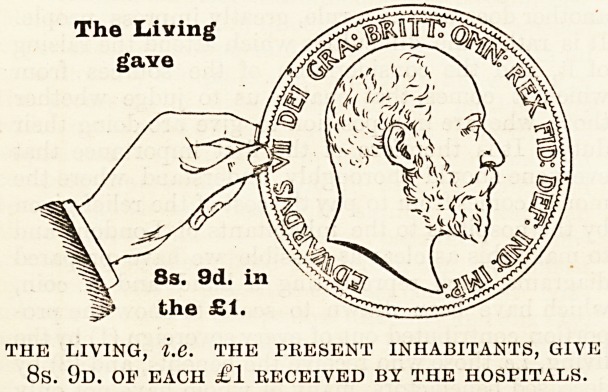


**Figure f3:**
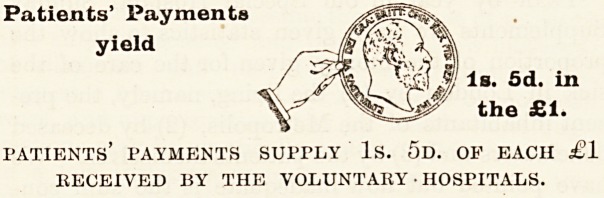


**Figure f4:**
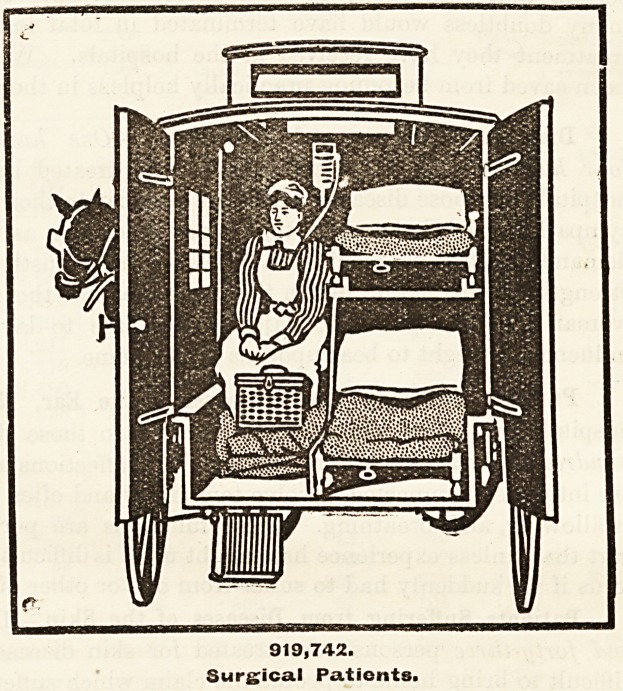


**Figure f5:**
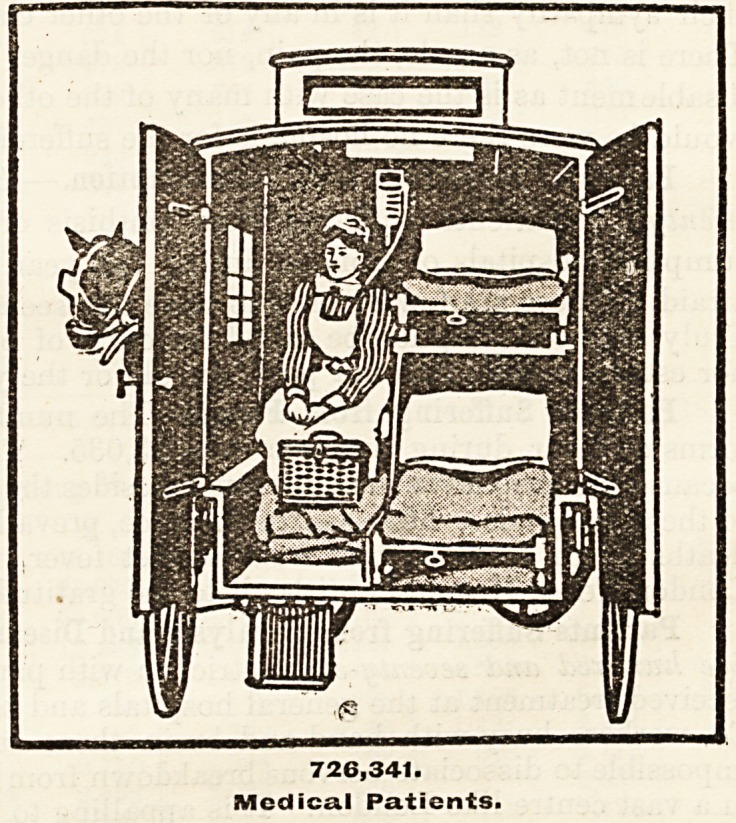


**Figure f6:**
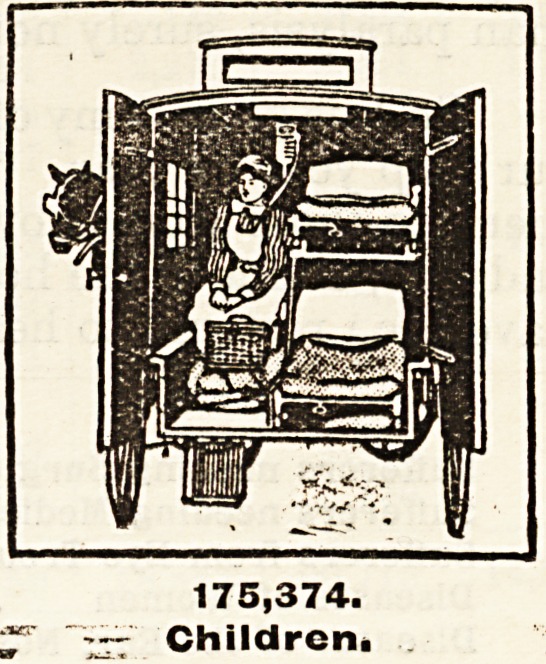


**Figure f7:**
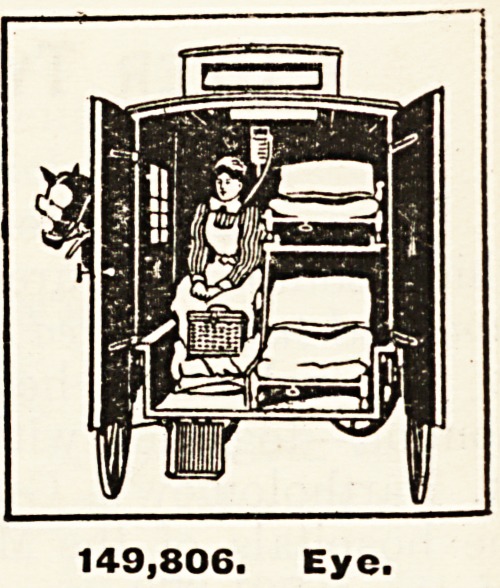


**Figure f8:**
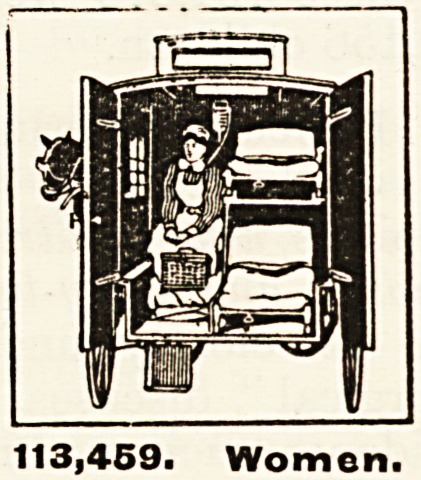


**Figure f9:**
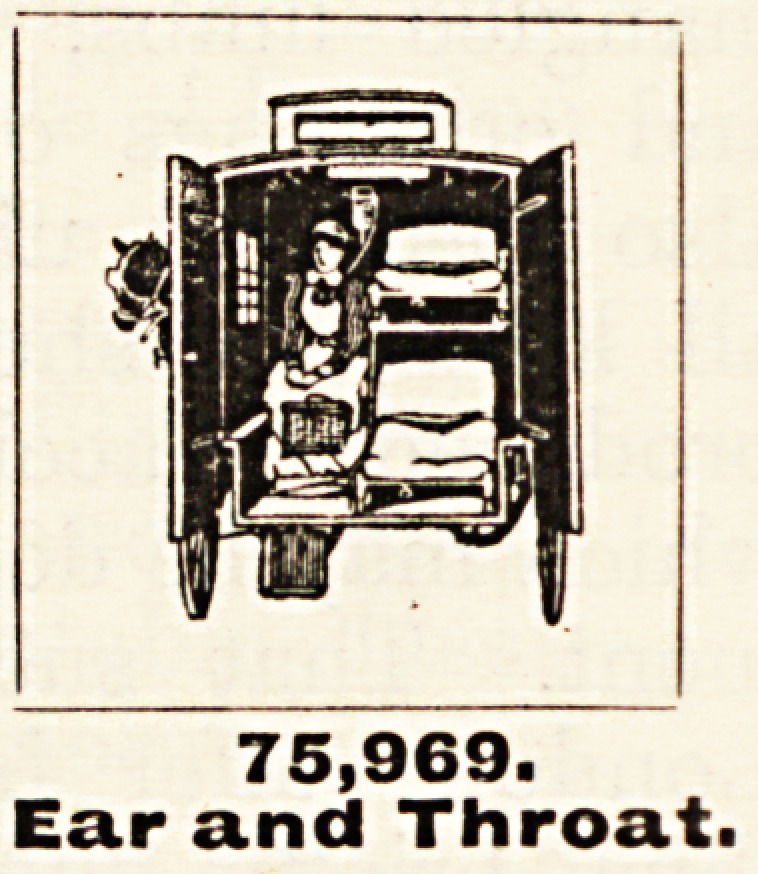


**Figure f10:**
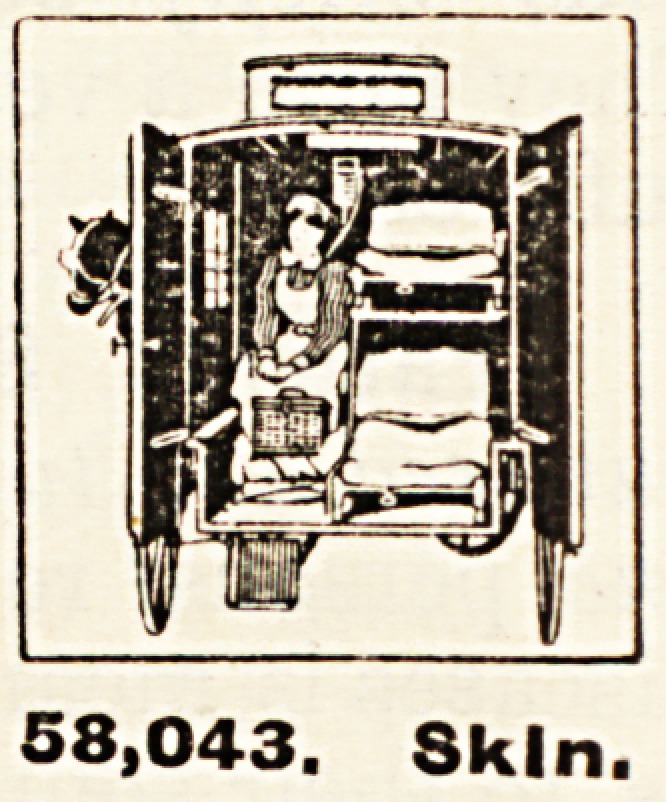


**Figure f11:**
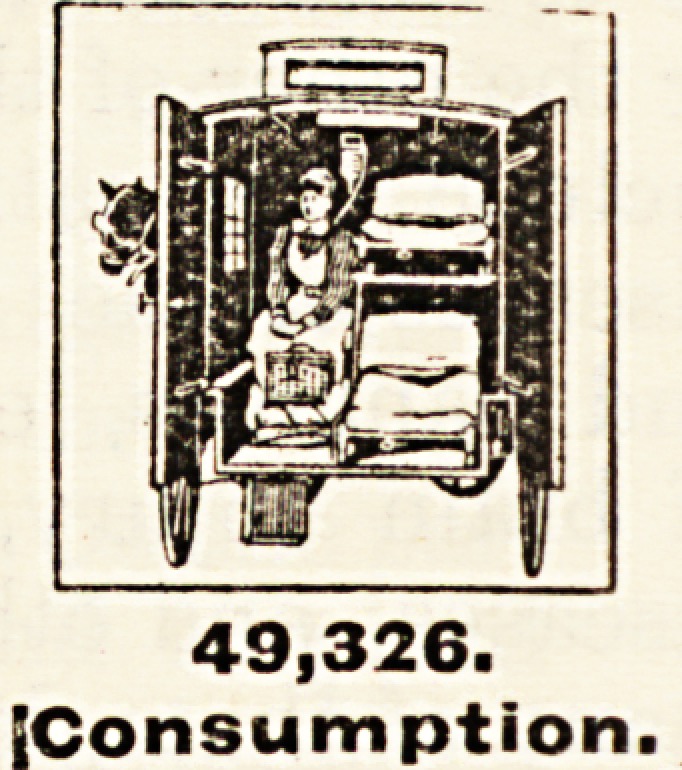


**Figure f12:**
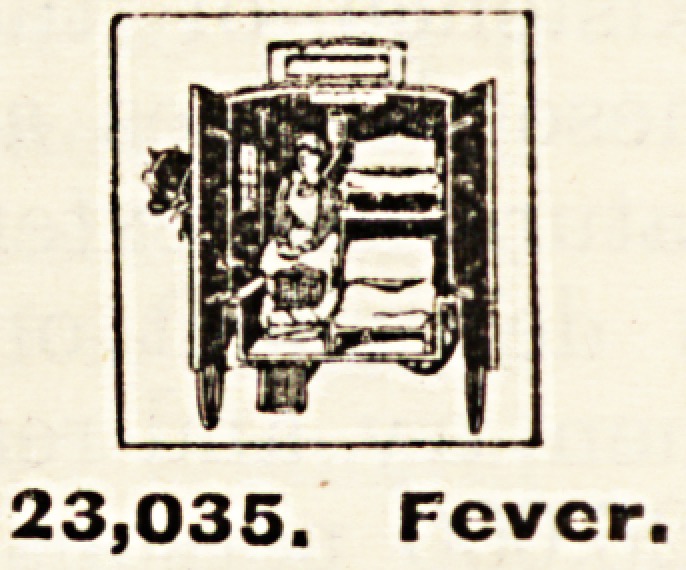


**Figure f13:**